# Editorial: TGF-β and T cell biology

**DOI:** 10.3389/fimmu.2023.1282656

**Published:** 2023-09-05

**Authors:** Saïdi Soudja, Nu Zhang

**Affiliations:** ^1^ Tumor Escape Resistance and Immunity Department, Cancer Research Center of Lyon (CRCL), INSERM U1052, CNRS UMR 5286, Centre Léon Bérard (CLB) and University of Lyon 1, Lyon, France; ^2^ Department of Microbiology, Immunology and Molecular Genetics, Long School of Medicine, University of Texas Health Science Center at San Antonio, San Antonio, TX, United States

**Keywords:** TGF-beta, T cells, exhaustion, Smad4, tissue residency, T cell differentiation

TGF-β is a versatile cytokine that plays a crucial role in immune regulation and maintaining overall homeostasis within the immune system (1). This multifaceted impact of TGF-β on T cells can be attributed to the cellular context, which means it might vary depending on the cell type and the state of activation or differentiation of the cells. Its actions can appear to be both pro-inflammatory and anti-inflammatory, depending on the context in which it is acting and the specific cell types involved. This complexity is essential for orchestrating appropriate immune responses while preventing excessive inflammation and immune-related disorders. This Research Topic presents reviews or original research articles that decorticate TGF-β effects on T cell biology and their physiological as well as pathological consequences.

TGF-beta (TGF-β) is a widely recognized factor that plays a crucial role in determining the tissue residency of CD8 T cells (2). The review of Ma and Zhang sheds light on how TGF-β intricately regulates the lymphoid tissue residency of TCF1+ exhausted T cells. These TCF1+ exhausted T cells are categorized as a progenitor or stem-like subset responsible for sustaining the entire pool of exhausted T cells (3). This particular subset constitutes a crucial target of checkpoint blockade immunotherapies, underlining their clinical significance. As these TCF1+ cells persist, they further differentiate, giving rise to transitional subsets and eventually to terminally exhausted T cells. The review contributes to a deeper understanding of how TGF-β governs the localization and differentiation of TCF1+ exhausted T cells within lymphoid tissues, highlighting its significant role in shaping the dynamics of exhausted T cell populations. TGF-β exerts its influence at multiple levels to determine the spatial trajectory of T cells. As highlighted in this review, TGF-β plays a pivotal role in the differentiation of TCF1+ stem-like cells, directing them toward residency in secondary lymphoid organs. In particular, although TGF-β promotes the generation of terminally differentiated exhausted T cells at later stage, it appears that TGF-β represses of the differentiation of TCF1+ stem like cells by in part enforcing their lymphoid tissue residency (4). This review exposes the dualistic impact of TGF-β on the fate of T cells. This intricate dichotomy likely arises from TGF-β’s ability to intervene at various stages of CD8 T cell differentiation, yielding distinct outcomes. When TGF-β acts upon TCF1+ stem-like cells, it fosters their residency potential while curtailing their propensity for terminal exhaustion. Conversely, at a later developmental stage, TGF-β could tilt the balance in favor of driving CD8 T cells toward terminal exhaustion, assuming a linear trajectory of differentiation. However, one can also envisage that TGF-β could exhibit distinct effect depending of the specific progenitor subsets, especially if the trajectory of differentiation follows a nonlinear pattern. This review pinpoints the dynamic nature of responses of TGF-β across different stages that govern their spatial and exhaustion status.

In alignment with this perspective, Chandiran et al. offers comprehensive insights into how TGF-β profoundly shapes all stages of CD8 T cell response. In particular, this review highlights chemokines, chemokine receptors and adhesion molecules controlled by TGF-β. Indeed, at different level TGF-β control migratory properties of CD8 T cells since naïve state by affecting the expression of these molecules regulating their migratory feature. The review of Chandiran et al. reminds us how TGF-β could affect CD8 biology since their naïve state to their final differentiated state. For instance, naïve CD8 T cells are preconditioned to become tissue resident memory CD8 T cells during interaction with migratory DCs that provide active TGF-β to naïve CD8 T cells in the secondary lymph node dictating their future spatial positioning (5). Along this line it has been also propose that SMAD4 preconditions naïve CD8 T cell fate in opposite way of TGF-β, revealing that SMAD4 and TGF-β are architects of inverse transcriptomic and epigenetic programs that guide the determination of CD8 T cells fate (6, 7). These insights further reinforce the complexity of TGF-β pathways in T cell biology and demand further investigations.

The complexity of TGF-β action can be amplified by integrating other regulatory pathways. Indeed some microRNAs play critical role in regulating TGF-β effects (8). The study of Kiran et al. was focused on role and mechanism of miR-10a-3p in adipose inflammation and adipogenesis. Their major finding is that miR-10a-3p modulates adiposity and suppresses inflammation through TGF-β1/Smad signaling pathway. The study shows distinct action of this microRNA depending of the cells. For instance, in immune cells, it seems to reduce the low grade of inflammation associated with condition of high fat diet and in adipocytes, it reduces lipid accumulation. The authors suggest that this reduced lipid accumulation goes through TGF-β/SMAD3 signaling although they did not formally show it. Overall, this study pinpoints an original mechanism by which TGF-β/signaling could be controlled in particular via microRNA with consequences in obesity.

The complexity of TGF-β action can be also generated by cooperating with other cytokines dictating the fate of the T cells. For instance, TGF-β acts synergistically with IL-6 to promote Th17 generation (1). Besides, TGF-β and IL-2 are critical players in the regulation of regulatory T cells (Treg). The concerted actions of TGF-β and IL-2 are essential for the development, survival, and suppressive functions of Tregs, contributing to immune homeostasis (1). Freudenberg et al. exemplify how TGF-β and IL-2 work together to promote Foxp3 lineage stability that could be enhanced with an inhibitor of DNA methylation. It appears that TGF-β-driven Foxp3 induction is not sufficient to fully recapitulate the epigenetic and transcriptional signature of *in vivo*-induced Foxp3+ Treg cells, including the failure to imprint Treg with a stable Foxp3 expression (9). The stabilization of Foxp3 expression can be overcome by pharmacological interference with DNA methyltransferase activity and CpG methylation (e.g., by cytosine nucleotide analogue). In this paper, Freudenberg et al. suggest that the effect of inhibition of DNA methyltransferase that promotes stabilization of Treg phenotype is mediated by a synergistic action of TGF-β and IL2 signaling. Although this paper did not formally demonstrate that the action of the pharmacological inhibitor goes through demethylation of the CNS2 region of Foxp3 gene. Several studies demonstrated now that demethylation of CNS2 is pivotal for the stability of Treg lineage (9, 10).

The intricate signaling cascades of TGF-β also greatly contribute to its diverse biological functions, adding additional complexity. TGF-β signaling includes three main pathways: one involving SMAD4, one involving TRIM33 and one involving non-canonical pathways that do not rely on SMAD3 and SMAD2. These pathways can interact synergistically, oppose each other, compete and even inhibit one another (11). This renders the decryption of TGF-β action complicated. The review of Chandiran et al. sheds light on some aspect of this complicated signaling pathways. SMAD4 in CD8 T cells has been described with a function, which does not depend on TGF-β in T cells that has an important impact in their subsequent differentiation. As referenced in this review, some recent papers demonstrated that ablation of SMAD4 gives an impressive opposite effects not only at the transcriptomic level but also at the functional level and also at the epigenetic level (6, 7).

Overall, the articles in this Research Topic offers comprehensive insights on the effect of TGF-β on some aspect of T cell biology.

## Author contributions

SS: Validation, Writing – original draft, Writing – review & editing. NZ: Validation, Writing – review & editing.
